# Effect of gender differences on the regulation of renal ischemia-reperfusion-induced inflammation in mice

**DOI:** 10.3892/mmr.2014.2089

**Published:** 2014-03-28

**Authors:** KYUNG PYO KANG, JUNG EUN LEE, AE SIN LEE, YU JIN JUNG, DAL KIM, SIK LEE, HONG PIL HWANG, WON KIM, SUNG KWANG PARK

**Affiliations:** 1 Department of Internal Medicine, Research Institute of Clinical Medicine, Chonbuk National University Medical School, Jeonju-si, Jeollabuk-do 561-712, Republic of Korea; 2 Department of Surgery, Research Institute of Clinical Medicine, Chonbuk National University Medical School, Jeonju-si, Jeollabuk-do 561-712, Republic of Korea

**Keywords:** gender, disparity, acute kidney injury, inflammation

## Abstract

Inflammation is a key mediator of renal ischemia-reperfusion (IR) injury. Gender disparities have been reported in acute and chronic kidney disease. In particular, males are considered to be more susceptible to renal ischemic injury compared with females according to animal studies. The purpose of the present study was to investigate the effect of gender on the renal inflammatory response following acute renal IR injury in mice. Experiments were performed in male and female C57BL/6 mice. Two weeks prior to the study, castration or ovariectomy were performed and testosterone propionate (100 μg/kg) or 17β-estradiol (100 μg/kg) was injected. Acute kidney injury (AKI) was induced by bilateral clamping of the renal pedicle for 23 min. Histological examination, western blot analysis and quantitative polymerase chain reaction were performed. In the acute renal IR injury model, the female mice were more resistant to kidney injury compared with the male mice. However, castration of the male mice reduced the levels of IR-induced tubular injury and macrophage infiltration compared with those in the injured male mice. Supplementation of testosterone reversed this protective effect in the male AKI model. Depletion of estrogen in the female mice increased the levels of IR-induced tubular injury and macrophage infiltration compared with those in the injured female mice. However, supplementation of estrogen in the ovariectomized female mice attenuated the IR-induced tubular injury and reduced the levels of macrophage infiltration. The expression levels of inflammatory cytokines, including tumor necrosis factor-α, monocyte chemotactic protein-1, interferon-γ and chemokine (C-C motif) ligand 17, were elevated in the male AKI mice compared with those in the control male mice, and were attenuated by castration. Estrogen depletion in the female mice significantly increased the expression levels of the renal inflammatory cytokines compared with those in the injured female mice, and were attenuated by estrogen supplementation in the ovariectomized female mice. These results suggested that the male gender confers greater susceptibility to IR renal injury due to an enhanced inflammatory response.

## Introduction

Inflammation is a key mediator of renal ischemia-reperfusion (IR) injury (1). Following ischemic injury, vascular endothelial cells and/or tubular epithelial cells are morphologically or functionally changed. The endothelial and tubular cells of injured kidneys generate inflammatory mediators, including cytokines and chemokines, which contribute to the recruitment of inflammatory cells into the kidneys (2). These inflammatory responses to ischemic injury have an important role not only in renal damage, but also in the reparative process.

A factor affecting the inflammatory process is the hormonal environment, particularly the sex hormones. Sex hormones have been reported to have an important role in IR-induced inflammatory processes in the kidneys (3). Meta-analysis data have shown that males exhibit rapid progression of non-diabetic kidney diseases, including membranous nephropathy, immunoglobulin (Ig) A nephropathy and autosomal dominant polycystic kidney disease (4). Previous studies have demonstrated that testosterone has an important role in increasing the susceptibility to ischemic renal injury compared with those of the depletion of estrogen or the absence of male sex hormones, which induced a reduction in the levels of post-ischemic oxidative stress in the kidneys (5,6). By contrast, other experimental results suggest that estrogen has a protective effect in ischemic renal injury via suppression of endothelin-1 production and activation of the phopsphatidylinositol-3 kinase/protein kinase B signaling pathway (7,8). Therefore, these data present conflicting results in regard to the role that sex hormones have in the pathophysiology of acute kidney injury (AKI).

Based on the aforementioned information, the present study was designed to investigate how depletion and/or repletion of sex hormones affect the renal IR-induced inflammatory process in male and female mice.

## Materials and methods

### Animal experiment

Male and female C57BL/6 mice (four weeks old; weight, 14–16 g) were purchased from Orient Bio Inc. (Seoul, Korea) and maintained in a room under controlled temperature (23±1°C), humidity, lighting (12-h light/12-h dark cycle) and free access to water. The animal experimental protocol was reviewed and approved by the Institutional Animal Care and Use Committee of Chonbuk National University (Jeonju-si, Korea; approval no., CBU 2011-0028). The experimental groups consisted of male and female sham groups (n=10, each group), male and female AKI groups (n=10, each group), castrated male and ovariectomized female AKI groups (n=10, each group) as well as castrated males treated with testosterone propionate and ovariectomized females treated with 17β-estradiol AKI groups (n=10, each group). The castration or ovariectomy was performed two weeks prior to induction of renal IR injury. From the day following the castration or ovariectomy, testosterone propionate (100 μg/kg; Sigma-Aldrich, St. Louis, MO, USA) or 17β-estradiol (100 μg/kg; Sigma-Aldrich) was injected intramuscularly daily during experimental periods. Renal ischemia was induced by bilateral clamping of the renal pedicles with a microvascular clip for 23 min, and then circulation was restored by removing both clips (9). Following sacrifice of the animals by CO_2_ inhalation, the kidneys were harvested to evaluate changes in the renal injury and the degree of renal inflammation at 48 h after the IR injury.

### Renal function analysis

On the final experimental day, blood was collected from the mice by intracardiac puncture immediately after anesthesia with ketamine (100 mg/kg; Huons, Seoul, Korea) and xylazine (10 mg/kg; Bayer Korea, Seoul, Korea). The blood urea nitrogen (BUN) and creatinine levels were measured by an enzymatic colorimetric method using an automatic analyzer (Hitachi 7180; Hitachi, Ltd., Tokyo, Japan).

### Histological examination

The kidneys were fixed in 4% paraformaldehyde and embedded in paraffin. The tissue was cut into 5-μm sections and stained with periodic acid-Schiff. The renal tubular injury was assessed as previously described (9). The tubular injury was scored by estimating the percentage of tubules in the cortex or the outer medullar that exhibited epithelial necrosis or luminal necrotic debris and tubular dilatation in a high-power field under a light microscope (Zeiss Z1 microscope; Carl Zeiss, Göttingen, Germany), with the scores as follows: 0, none; 0.5, <10%; 1, 10–25%; 2, 25–50%; 3, 50–75%; and 4, >75%. All evaluations were made on 10 non-overlapping fields per section and 10 sections per kidney were analyzed. The morphometric examinations were performed in a blinded manner by two independent investigators.

### Immunofluorescence staining

Immunofluorescence staining was performed as described previously (10). Briefly, freshly frozen renal tissues were fixed with 4% paraformaldehyde, permeabilized in 1% Triton X-100 and then incubated with a blocking buffer. Subsequently, the samples were incubated with rat anti-mouse F4/80 antibody (eBioscience, Inc., San Diego, CA, USA). The slides were exposed to Cy3-labeled secondary antibody (Chemicon, Temecula, CA, USA). The nuclear staining was performed using DAPI. The digital images were captured with a Zeiss Z1 microscope (Carl Zeiss AG, Göttingen, Germany) in 10 randomly chosen, non-overlapping fields at a magnification of ×400.

### Quantitative polymerase chain reaction (qPCR)

qPCR was performed as described previously (11). Briefly, total RNA was extracted from the kidney homogenates using TRIzol^®^ reagent (Invitrogen, Carlsbad, CA, USA). A Transcriptor First Strand cDNA Synthesis Kit (Roche Diagnostics, Mannheim, Germany) was used to synthesize cDNA from the total RNA according to the manufacturer’s instructions. Specific primers for each gene (Table I) were designed using Primer Express software, v3.0 (Applied Biosystems, Inc., Foster City, CA, USA). qPCR was performed in a 7900HT Fast Real-Time PCR system (Applied Biosystems, Inc.). A 10-fold dilution of each cDNA transcript was amplified in a 10-μl volume, using SYBR^®^ Green PCR Master Mix (Applied Biosystems, Inc.), with a 200 nmol/l final concentration of each primer. To confirm the use of equal amounts of RNA in each reaction, all samples were examined in parallel for glyceraldehyde 3-phosphate dehydrogenase mRNA expression.

### Western blot analysis

Western blot analysis was performed as described previously (12). The kidney tissues were homogenized in phosphate-buffered saline with a protease inhibitor cocktail (Calbiochem, San Diego, CA, USA) and the protein concentration was quantified using the Bradford protein assay. The samples (40 μg protein per lane) were mixed with buffer, boiled for 6 min, separated by 8% SDS-PAGE and electroblotted onto a nitrocellulose membrane (Bio-Rad Laboratories, Inc., Hercules, CA, USA). The membrane was blocked with 5% nonfat dry milk in Tris-buffered saline with Tween-20 buffer (25 mmol/l Tris, pH 7.5, 150 mmol/l NaCl and 0.1% Tween-20) for 1 h and then incubated overnight at 4°C with goat anti-mouse intercellular adhesion molecule (ICAM)-1 monoclonal antibody (Santa Cruz Biotechnology, Inc., Santa Cruz, CA, USA). The blots were washed with Tris-buffered saline and Tween 20 buffer and incubated with horseradish peroxidase-conjugated donkey anti-goat IgG (Santa Cruz Biotechnology, Inc.). Signals were visualized with a chemiluminescent detection kit according to the manufacturer’s instructions (Amersham Pharmacia Biotech, London, UK). The membranes were then reprobed with a rabbit anti-mouse actin antibody to verify equal loadings of protein in each lane. All signals were analyzed by densitometric scanning (LAS-3000; Fujifilm, Tokyo, Japan).

### Statistical analysis

Data are expressed as the mean ± standard deviation. Multiple comparisons were examined for significant differences using analysis of variance, followed by individual comparison with the Tukey’s post-hoc test, with P<0.05 considered to indicate a statistically significant difference.

## Results

### Effect of gender differences on renal function and histological changes following IR injury

**Figure 1:**
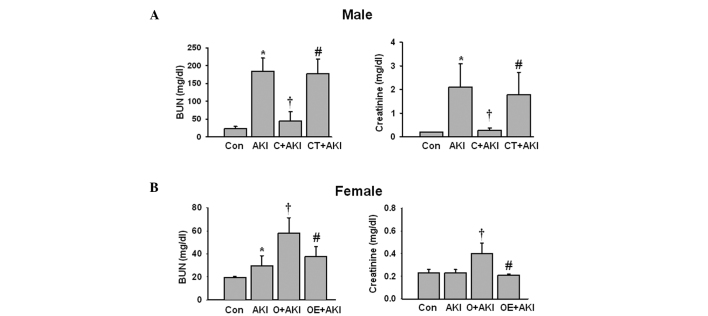
Effect of sex hormones on renal function following IR injury. AKI was induced by bilateral renal ischemia for 23 min. Blood samples were collected 48 h after IR injury from the normal male mice (Con), normal male mice with AKI (AKI), castrated male mice with AKI (C+AKI), testosterone-replaced castrated male mice with AKI (CT+AKI), as well as normal female mice (Con), normal female mice with AKI (AKI), ovariectomized female mice with AKI (O+AKI), and estrogen-replaced ovariectomized female mice with AKI (OE+AKI). The BUN and creatinine levels from (A) male and (B) female mice were measured. Data are expressed as the mean ± standard deviation (n=10 mice per group). ^*^P<0.05 vs. Con; ^†^P<0.05 vs. AKI; ^#^P<0.05 vs. castrated or ovariectomized AKI. BUN, blood urea nitrogen; IR, ischemia-reperfusion; AKI, acute kidney injury.

The male mice exhibited significantly elevated BUN and creatinine levels following IR injury compared with those of the male control group. Following castration, renal function was preserved despite the ischemic injury. However, testosterone replacement in the castrated male mice aggravated renal function following IR injury compared with that of the castrated male AKI group (Fig. 1A). In the female mice, IR injury induced a minimal significant increase in the levels of BUN compared with those of the control female mice. The levels of creatinine did not change significantly in the female mice with AKI compared with those of the control female mice. However, the ovariectomized female mice exhibited significantly increased BUN and creatinine levels compared with those of the mice in the female AKI group. Replacement of estrogen in the ovariectomized female mice attenuated the IR-induced increase in the BUN and creatinine levels compared with those of the ovariectomized female mice (Fig. 1B).

### Tubular injury score increases significantly in the male AKI group compared with that of the control group

**Figure 2:**
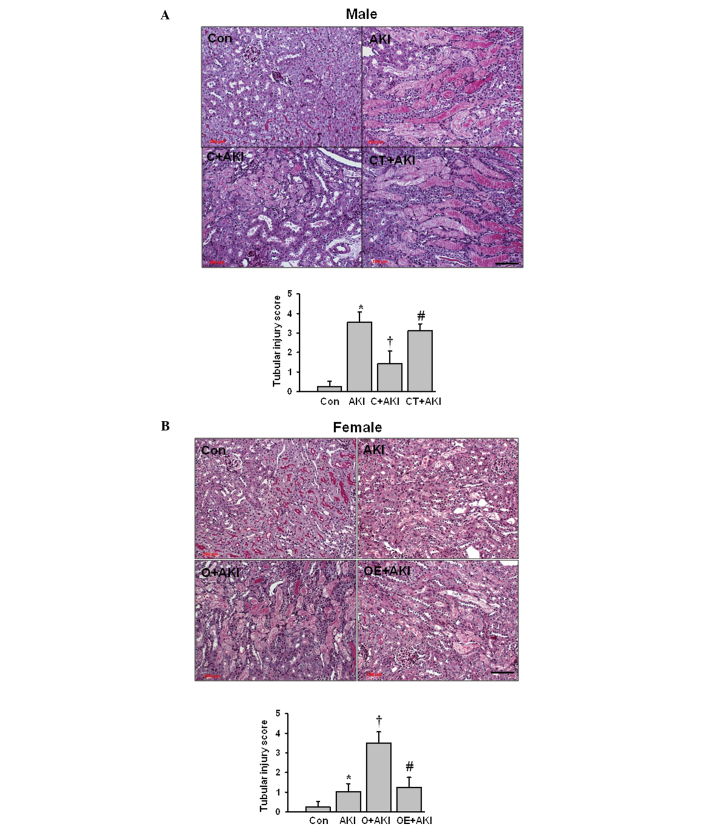
Renal histological findings following IR injury. Representative histological sections of kidney from (A) normal male mice (Con), normal male mice with AKI (AKI), castrated male mice with AKI (C+AKI) and testosterone-replaced castrated male mice with AKI (CT+AKI) as well as (B) normal female mice (Con), normal female mice with AKI (AKI), ovariectomized female mice with AKI (O+AKI) and estrogen-replaced ovariectomized female mice with AKI (OE+AKI). Semiquantitative scoring of the tubular injury was concomitant with histological analysis. Data are expressed as the mean ± standard deviation (n=10 mice per group). ^*^P<0.05 vs. Con; ^†^P<0.05 vs. AKI; ^#^P<0.05 vs. castrated or ovariectomized AKI. IR, ischemia-reperfusion; AKI, acute kidney injury. Staining, periodic acid-Schiff; bar, 100 μm.

Following castration, the tubular injury score decreased significantly when compared with that of the male AKI group. However, replacement of testosterone in the castrated male mice aggravated the tubular injury compared with that in the mice in the castrated male AKI group (Fig. 2A). The ovariectomized female mice had significantly increased levels of tubular injury compared with those of the female AKI group. Estrogen replacement in the ovariectomized female mice significantly attenuated the IR-induced tubular damage compared with that in the ovariectomized female AKI group (Fig. 2B).

### Effect of gender differences on the levels of macrophage infiltration following IR injury

**Figure 3:**
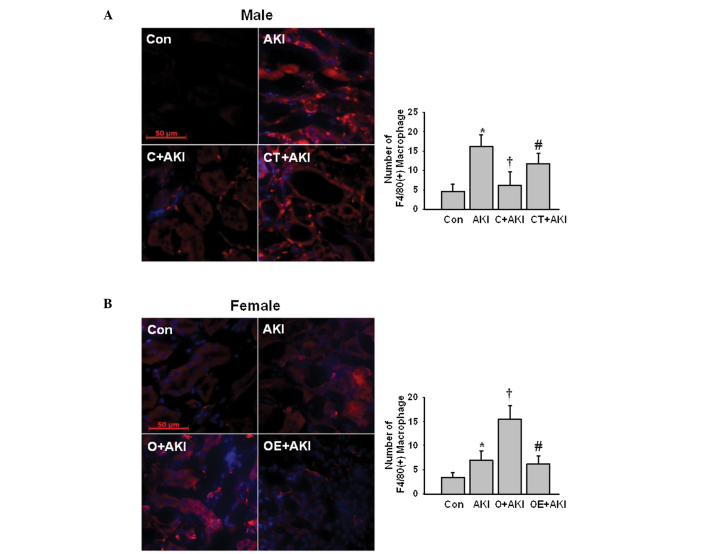
Effect of sex hormones on F4/80(+) macrophage infiltration following IR injury. Representative immunofluorescence staining for F4/80 in kidneys from (A) normal male mice (Con), normal male mice with AKI (AKI), castrated male mice with AKI (C+AKI) and testosterone-replaced castrated male mice with AKI (CT+AKI) as well as (B) normal female mice (Con), normal female mice with AKI (AKI), ovariectomized female mice with AKI (O+AKI) and estrogen-replaced ovariectomized female mice with AKI (OE+AKI). Number of F4/80(+) macrophages per ×400 magnification. Bars represent the mean ± standard deviation (n=10 for each experimental group). ^*^P<0.05 vs. Con; ^†^P<0.05 vs. AKI; ^#^P<0.05 vs. castrated or ovariectomized AKI. IR, ischemia-reperfusion; AKI, acute kidney injury.

The F4/80(+) macrophage infiltration was evaluated following IR injury in the male and female mice. The number of F4/80(+) macrophages increased significantly following IR injury in the male AKI group compared with that in the male control group. Following castration, the levels of F/80(+) macrophage infiltration were significantly reduced compared with those in the male AKI group. However, replacement of testosterone in the castrated male mice significantly increased the levels of F4/80(+) macrophage infiltration following IR injury compared with those in the castrated male mice (Fig. 3A). In the ovariectomized female mice, the number of F4/80(+) macrophages increased following IR injury compared with that in the female AKI group. The replacement of estrogen attenuated the IR-induced increase in the levels of F4/80(+) macrophage infiltration compared with those in the ovariectomized female AKI group (Fig. 3B).

### Effect of gender differences on renal cytokine expression levels following IR injury

**Figure 4:**
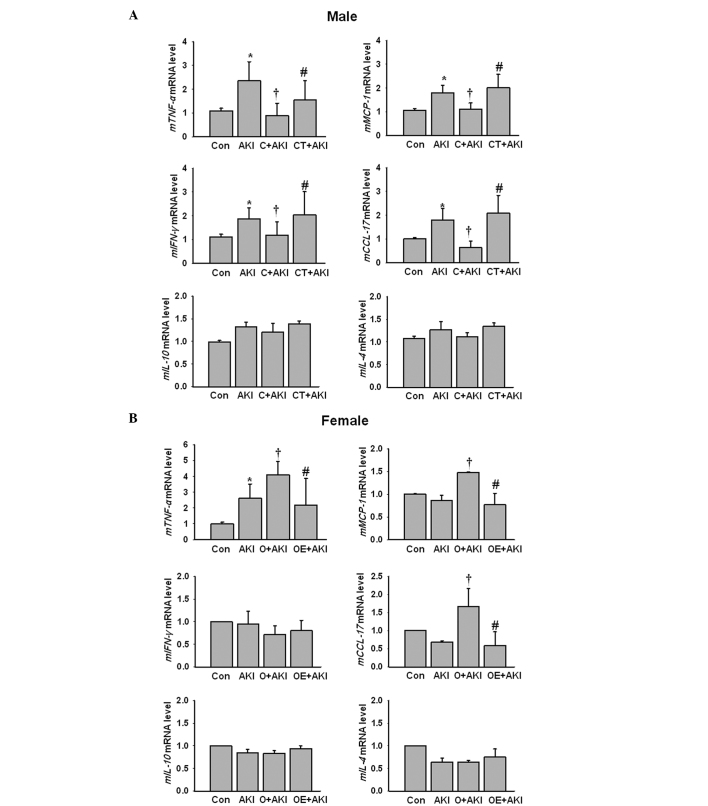
Effect of sex hormones on cytokine expression levels following IR injury. Kidneys from (A) normal male mice (Con), normal male mice with AKI (AKI), castrated male mice with AKI (C+AKI) and testosterone-replaced castrated male mice with AKI (CT+AKI) as well as (B) normal female mice (Con), normal female mice with AKI (AKI), ovariectomized female mice with AKI (O+AKI) and estrogen replaced ovariectomized female mice with AKI (OE+AKI) were evaluated for the levels of TNF-α, MCP-1, IFN-γ, CCL-17, IL-10, and IL-4 mRNA expression by quantitative polymerase chain reaction. Data are presented as the relative expression levels to those of the control after normalization with GADPH. Bars present the mean ± standard deviation (n=10 for each experimental group). ^*^P<0.05 vs. Con; ^†^P<0.05 vs. AKI; ^#^P<0.05 vs. castrated or ovariectomized AKI. TNF, tumor necrosis factor; MCP, monocyte chemotactic protein; IFN, interferon; CCL, chemokine (C-C motif) ligand; IL, interleukin; IR, ischemia-reperfusion; AKI, acute kidney injury.

Subsequently, the cytokine and chemokine expression levels were evaluated following IR injury using qPCR in the male and female mice. Tumor necrosis factor (TNF)-α, monocyte chemotactic protein (MCP)-1, interferon (IFN)-γ and chemokine (C-C motif) ligand (CCL)-17 mRNA levels were significantly increased following IR injury in the male AKI group compared with those in the male control group. Depletion of testosterone by castration reduced the expression levels of TNF-α, MCP-1, IFN-γ and CCL-17 mRNA compared with those in the male AKI group. Following replacement of testosterone in the castrated male mice, the expression levels of the proinflammatory cytokines reversed compared with those in the castrated male mice. The expression levels of interleukin (IL)-10 and IL-4 mRNA following renal IR injury did not change significantly between the male groups despite hormonal modulation in the male mice (Fig. 4A). In female mice, the TNF-α mRNA levels were significantly increased following IR injury compared with those in the control female group. Following estrogen depletion via ovariectomy, the expression levels of TNF-α, MCP-1 and CCL-17 mRNA increased significantly compared with those in the female AKI group. However, following the replacement of estrogen in the ovariectomized female mice, the expression levels of the proinflammatory cytokines, with the exception of IFN-γ, decreased significantly compared with those in the ovariectomized AKI group. The expression levels of IL-10 and IL-4 mRNA did not change significantly following AKI in the female mice compared with those in the control female group (Fig. 4B).

### Effect of gender differences on renal intercellular adhesion molecule (ICAM)-1 expression levels following IR injury

**Figure 5:**
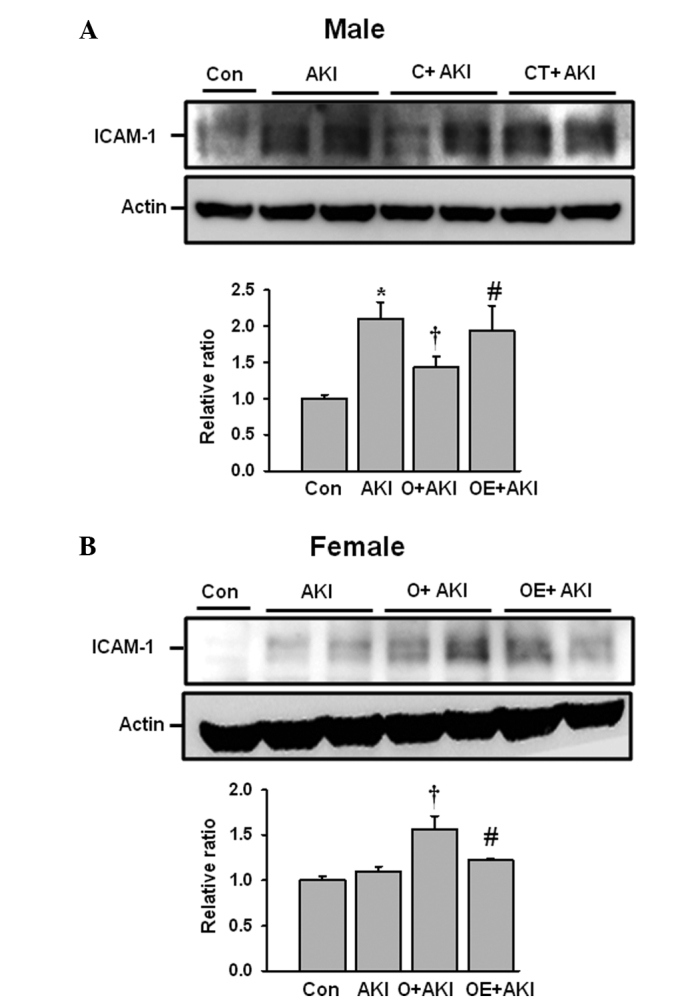
Effect of sex hormones on ICAM-1 expression levels following IR injury. Kidneys from (A) normal male mice (Con), normal male mice with AKI (AKI), castrated male mice with AKI (C+AKI) and testosterone-replaced castrated male mice with AKI (CT+AKI) as well as normal female mice (Con), normal female mice with AKI (AKI), ovariectomized female mice with AKI (O+AKI) and estrogen-replaced ovariectomized female mice with AKI (OE+AKI) were evaluated for the ICAM-1 expression levels by western blot analysis. The relative ratio measured in the kidneys from the control mice is arbitrarily presented as one. Bars represent the mean ± standard deviation (n=10 for each experimental group). ^*^P<0.05 vs. Con; ^†^P<0.05 vs. AKI; ^#^P<0.05 vs. castrated or ovariectomized AKI. ICAM, intercellular adhesion molecule; IR, ischemia-reperfusion; AKI, acute kidney injury.

The expression levels of ICAM-1 were also evaluated following IR injury in the male and female mice. The male mice in the AKI group exhibited significantly increased ICAM-1 expression levels compared with those in the control group. Following castration, the ICAM-1 levels decreased compared with those in the AKI group. Replacement of testosterone in the castrated male mice significantly increased the levels of ICAM-1 expression compared with those in the mice in the castrated male AKI group (Fig. 5A). The ovariectomized female group had significantly increased ICAM-1 expression levels following IR injury compared with those in the female AKI group. However, estrogen replacement reduced the ICAM-1 expression levels following IR injury (Fig. 5B).

## Discussion

The present study demonstrated that male and female mice exhibit different inflammatory responses following IR injury. Testosterone depletion in the male mice attenuated the renal IR-induced inflammatory response compared with that in the male mice with AKI; however, estrogen depletion in the female mice aggravated the inflammatory response compared with that in the female mice with AKI. The IR-induced renal inflammatory responses were more prominent in the castrated male mice treated with testosterone replacement compared with those in the castrated male mice without hormone replacement. Estrogen replacement in the ovariectomized female mice recovered the attenuated IR-induced renal inflammatory response compared with that in the female mice in an estrogen-depleted state.

The renal inflammatory response is responsible for the pathogenesis of acute and chronic kidney injury. Inflammatory cytokines or chemokines are produced due to the renal IR injury and have an important role in the recruitment of inflammatory cells, including neutrophils and monocytes/macrophages (13). Several studies suggest that gender- or sex hormone-mediated inflammatory processes are responsible for ischemic injury processes in the liver (14), brain (15) and heart (16). Similar findings have been demonstrated in renal IR injury (5,6). Consistent with the findings of previous studies, the present study identified that female mice were more resistant to renal IR injury compared with male mice. However, replacement of estrogen in the ovariectomized female mice did not result in a completely protective effect following IR injury compared with that in the mice with AKI. The female mice exhibited an increase in the TNF-α mRNA expression levels following renal IR injury, despite no change in the levels of MCP-1, IFN-γ and CCL17 mRNA expression following IR injury compared with those in the control female mice. These data suggest that estrogen exerts a partially protective effect on the renal IR injury-induced inflammatory response. In contrast to that of female sex hormones, testosterone depletion is more protective in renal IR injury.

Suppression of the inflammatory response following renal IR injury preserves renal function and prevents progression to chronic kidney disease. The present study identified that the sex hormone status may be important for initiation of the inflammatory response following renal IR injury. In the male mice, the proinflammatory cytokine expression levels increased significantly following renal IR injury compared with those in the control mice. However, depletion of testosterone reduced the expression levels of the proinflammatory cytokines compared with those in the mice with AKI. Furthermore, testosterone replacement in the castrated male mice aggravated the IR-induced renal inflammatory response compared with that in the castrated mice without hormone replacement. In the female mice, these inflammatory responses were less prominent even following estrogen depletion or replacement of estrogen in the ovariectomized female mice. These data suggest that testosterone may have more important roles than estrogen in renal IR injury via enhancing the renal inflammatory response.

In conclusion, the results of the present study suggested that the male gender confers greater susceptibility to renal IR injury via the enhancement the inflammatory response by testosterone. Further studies are required to address the underlying mechanism of the gender differences in AKI.

## Figures and Tables

**Table I:** Sequences and accession numbers of the forward and reverse primers used in the quantitative polymerase chain reaction.

Gene	Accession no.	Forward primer sequence	Reverse primer sequence
TNF-α	(NM 013693)	AGGGTCTGGGCCATAGAACT	CCACCACGCTCTTCTGTCTAC
MCP-1	(NM 011333)	ATTGGGATCATCTTGCTGGT	CCTGCTGTTCACAGTTGCC
IFN-γ	(NM 008337)	TGAGCTCATTGAATGCTTGG	ACAGCAAGGCGAAAAAGGAT
CCL-17	(NM 011332)	ATAGGAATGGCCCCTTTGAA	TGCTTCTGGGGACTTTTCTG
IL-10	(NM 010548)	TGTCAAATTCATTCATGGCCT	ATCGATTTCTCCCCTGTGAA
IL-4	(NM 021283)	CGAGCTCACTCTCTGTGGTG	TGAACGAGGTCACAGGAGAA
GAPDH		TTGAGGTCAATGAAGGGGTC	TCGTCCCGTAGACAAAATGG

TNF, tumor necrosis factor; MCP, monocyte chemotactic protein; IFN, interferon; IL, interleukin; CCL, chemokine (C-C motif) ligand.
